# Emodin Improves Intestinal Health and Immunity through Modulation of Gut Microbiota in Mice Infected by Pathogenic *Escherichia coli* O_1_

**DOI:** 10.3390/ani11113314

**Published:** 2021-11-19

**Authors:** Ruijuan Gao, Chunjie Wang, Aricha Han, Yanping Tian, Shunan Ren, Wenting Lv, Aorigele Chen, Jian Zhang

**Affiliations:** 1 College of Veterinary Medicine, Inner Mongolia Agricultural University, Hohhot 010018, China; Gaoruijuan01@126.com (R.G.); Tianyanping74823@163.com (Y.T.); shunan_ren@sina.com (S.R.); ccmm0508@yeah.net (W.L.); 2 College of Animal Science, Inner Mongolia Agricultural University, Hohhot 010018, China; 15248146527@163.com (A.H.); aori6009@163.com (A.C.); ZhangJian280613@163.com (J.Z.)

**Keywords:** emodin, *Escherichia coli* O_1_, diarrhea, intestinal mucosal barrier, 16S rRNA sequencing

## Abstract

**Simple Summary:**

Diarrhea is associated with intestinal inflammation and ecological imbalance of the intestinal flora. Emodin is an herbal ingredient with various pharmacological effects, such as antibacterial, anti-inflammatory, and anti-cancer. Although several characteristics of emodin have been reported, the effects of orally administered emodin on the structure of the intestinal microbial community and immunological parameters in mice with enterotoxigenic *Escherichia coli* (ETEC) infection are not well understood. Therefore, this study focused on determining whether emodin mitigates the negative effects of ETEC infection on intestinal barrier function and immune response in mice by altering intestinal flora. The results suggest that emodin protects against intestinal damage induced by *E. coli* O_1_ and improves intestinal mucosal barrier function in mice by increasing the abundance of beneficial intestinal microbiota and inhibiting the abundance of harmful bacteria, thereby alleviating diarrhea. The findings of this study will contribute to our understanding of how emodin attenuates the effects of ETEC on intestinal mucosal barrier damage by modulating the microbial composition of the gut.

**Abstract:**

The effect of emodin on the intestinal mucosal barrier of a mouse *E. coli* O_1_-induced diarrhea model was observed. Following successful establishment of a diarrhea model, the mice were treated with drugs for seven days. Intestinal lesions and the shape and the number of goblet cells were assessed via hematoxylin-eosin and periodic-acid-Schiff staining, while changes in inflammatory factors, ultrastructure of the small intestine, expression of MUC-2, and changes in the intestinal microbiota were analyzed via RT-PCR, electron microscopy, immunofluorescence, and 16S rRNA sequencing. Examination showed that emodin ameliorated pathological damage to the intestines of diarrheic mice. RT-PCR indicated that emodin reduced TNF-α, IL-β, IL-6, MPO, and COX-2 mRNA levels in duodenal tissues and increased the levels of sIgA and MUC-2 and the number of goblet cells. Microbiome analysis revealed that *Escherichia coli* O_1_ reduced bacterial richness and altered the distribution pattern of bacterial communities at the phylum and order levels in cecum contents. Notably, pathogenic *Clostridiales* and *Enterobacteriales* were significantly increased in diarrheic mice. However, emodin reversed the trend. Thus, emodin protected against intestinal damage induced by *E. coli* O_1_ and improved intestinal mucosal barrier function in mice by increasing the abundance of beneficial intestinal microbiota and inhibiting the abundance of harmful bacteria, thereby alleviating diarrhea.

## 1. Introduction

Intestinal microbiota regulate and maintain the normal physiological functions of the body such as digestion [1], metabolism [2,3], and immunity [4,5]. Alterations in the composition of the gut microbiota can lead to a variety of diseases and dysfunctions, including inflammatory bowel disease [6], obesity [7], colorectal cancer [8], and type 2 diabetes [9]. There is a strong link between the host’s gut health and the gut microbiota. Alterations in the composition of the gut microbiota can disrupt the intrinsic and adaptive defenses mediated by it, thereby increasing the susceptibility of the gut to infection. However, pathogenic infections of the gut can also affect the composition of the intestinal microbiota [10]. For example, *Escherichia coli* O_101_ infection decreased the abundance of firmicutes and verrucomicrobia and increased the abundance of proteobacteria and actinobacteria [11]. In addition, the metabolites of the intestinal flora play a crucial role in regulating the physiological functions of the host. For instance, the digestive products of *Butyricicoccus* are able to produce butyric acid, which improves intestinal permeability [12]. Thus, changes in the gut microbiota may be relevant to the pathogenesis of infectious diseases.

Enterotoxigenic *Escherichia coli* (ETEC) infection causes diarrhea, disrupts intestinal microbiota balance, damages normal intestinal morphology, and triggers an inflammatory response, resulting in decreased growth rates in young animals with significant economic losses [13,14]. ETEC is also the main infectious agent responsible for diarrheic diseases in young animals including piglet dysentery, swine edema, chicken dysentery, and calf diarrhea, among others [15]. Fifty percent of annual piglet deaths worldwide are attributed to ETEC [16]. ETEC alters the barrier function of the intestinal epithelium by adhering to the small intestinal epithelium and secreting enterotoxins, thereby triggering diarrhea [17]. Studies have indicated that ETEC damages the intestinal barrier by activating the NF-κB and MAPK pathways and promoting the expression of pro-inflammatory cytokines [18]. ETEC infection produces enterotoxins that disturb intestinal microbiota and upregulate IL-17 expression, leading to diarrhea [19,20].

Currently, ETEC related diarrhea is being widely treated with therapeutic antibiotics or antimicrobial growth promoters. However, the available evidence suggests that clinical misuse or inappropriate use of antibiotics leads to the emergence of antibiotic-resistant microorganisms, poor adherence, and increased morbidity associated with antibiotic use [21]. Thus, effective prevention and treatment of *E. coli* related diarrhea, as well as the development of new alternative medicines to replace antibiotics that are currently being used, is increasingly gaining the attention of those engaged in veterinary science.

Among non-antibiotic alternatives, traditional medicines based on plant-derived molecules or extracts have the highest potential, as these are effective against diseases on account of their immunostimulatory, anti-inflammatory, antiviral, anticancer, and radioprotective properties [15,22]. Emodin is a plant extract which is widely used in traditional Chinese medicine and may be extracted from various plants such as *Cassia obtusifolia*, *Aloe vera*, *Polygonum multiflorum*, and *Polygonum cuspidatum* [23]. It exerts numerous beneficial effects, such as the suppression of apoptosis in T cells [24] and regulation of intestinal microbiota [25], as well as antibacterial [26], anti-inflammatory [27], and anticancer effects [28] in humans and animals. Ning et al., experimentally illustrated that emodin, which decreased intestinal mucosal damage in severe acute pancreatitis was associated with decreased levels of (Interleukin 1β) IL-1β and (Interleukin 18) IL-18 [29]. Li et al., demonstrated that emodin protected intestinal barrier integrity in rat cecal ligation and puncture models by increasing the expression of tight junction (TJ) proteins, claudin-3, ZO-1, and occludin [30]. Although many features of emodin have been reported, the effects of orally administered emodin on the structure of the intestinal microbial community and immunological parameters in mice under ETEC stimulation have rarely been reported.

Therefore, we used mouse ETEC-infected specific-pathogen-free models to determine whether emodin ameliorates the negative effects of ETEC infection on intestinal barrier function and immune response in mice by altering intestinal microbiota [13].

## 2. Materials and Methods

### 2.1. Bacterial Culture and Diarrhea Modelling

*E. coli* O_1_ was sequentially cultured in eosin-methylene blue medium, MacConkey medium (MAC), and nutritional broth medium, and then incubated at 37 °C to log phase for 18–24 h. The bacterial suspension was diluted with physiological saline to achieve a bacterial concentration of 2.5 × 10^11^ CFU/mL. *E.* *coli* O_1_ was provided by Professor Aorigele Chen, from the cattle production laboratory of the College of Animal Science of Inner Mongolia Agricultural University.

Diarrhea was induced with intragastric administration of *E. coli* O_1_ (0.3 mL of 2.5 × 10^11^ CFU/mL) [31] for three consecutive days as described previously. The typical diarrhea symptoms of mice, such as disheveled hair, slow movement, and poor mental state, were used as indicators to show success in modeling. Treatment with emodin and ciprofloxacin by oral gavage twice daily for 1 week started 12 h after diarrhea induction.

### 2.2. Animals and Trial Groups

The mice were housed in ventilated cages under light/dark cycle for 12 h, room temperature 21 ± 1 °C with free access to standard experimental food and water, and a mouse model of *E. coli* O_1_-induced diarrhea was established after 72 h of adaptation. The study included 80 specified pathogen free (SPF) Kunming mice (aged 30 days, weighing 20 ± 2 g), 40 male and 40 female, which were acquired from the Laboratory Animal Center of Inner Mongolia University (License No: Scxk (Meng) 2016--0001). The study complied with rules approved by the Institutional Animal Care and Use Committee of Inner Mongolia Agricultural University (reference no. 20190917).

Diarrhea model mice were divided randomly into a vehicle group (VG, *n* = 10, 5M/5F); a model group (MG, *n* = 10, 5M/5F); a ciprofloxacin group (CG, *n* = 10, 5M/5F); a low-dose emodin group (Em-L, *n* = 10, 5M/5F); a middle-dose emodin group (Em-M, *n* = 10, 5M/5F); and a high-dose emodin group (Em-H, *n* = 10, 5M/5F). The MG and CG groups were administered saline and 0.13 mg/kg ciprofloxacin, respectively, by oral gavage; the Em-L, Em-M, and Em-H groups were administered 8.75, 17.5, and 35 mg/kg emodin dissolved in a 0.5% sodium carboxy-methyl-cellulose solution (prepared to contain emodin at 5 g/L), respectively, by oral gavage, once daily. The emodin doses were determined based on the LD50 [32].

### 2.3. Sample Collection

Six hours after the final administration, the mice were euthanized under anesthesia by exsanguination. Subsequently, 2-cm-long duodenum of each sample were quickly dissected out under sterile conditions and rinsed with ice saline. One of these tissue sections was immediately stored in formalin and 2.5% glutaraldehyde (pH 7.4, 4 °C) for hematoxylin-eosin (HE) staining, Periodic acid–Schiff (PAS) staining, transmission electron microscopy experiments, and immunofluorescence experiments. Other duodenum tissue and contents of the cecum were obtained in sterile enzyme-free lyophilization tubes, stored overnight in liquid nitrogen, and transferred to −80 °C for storage for cytokine gene expression detection and 16s high-throughput sequencing experiments.

### 2.4. Histopathological Examination

One week after fixation, intestinal tissue samples were rinsed with slow running water for 12 h (water temperature: 14 °C), dehydrated in an ethanol gradient (70% for 1 h, 80% for 1 h, 90% for 1 h, 95% for 40 min, 100% for 40 min), submersed in xylene for 40 min, then in xylene/paraffin wax (1:1 by volume, 1 h), and then paraffin wax for 2–3 h before being embedded into hard wax (melting point: 58–60 °C). Serial slices of 5 μm thickness were then cut on a microtome. After HE staining, histopathological changes of the duodenum (*n* = 8) were observed at 200× total magnification using an X-Cite120Q wide field fluorescence microscope (Lumen Dynamics, Mississauga, ON, Canada). The degree of damage to intestinal tissue was graded on a 0–5 scale according to Chiu’s scale, as follows: normal villi = 0, edema at the tip of the villi = 1, dilated subepithelial spaces in the mucosa, and detachment of mucosal epithelium from the lamina propria = 2; massive detachment of epithelial cells from the lamina propria, and partial detachment of the ends of the villi = 3; detachment of intestinal villi, lamina propria, and capillaries dilatation = 4; bleeding, and ulceration of the lamina propria of the intestine = 5.

### 2.5. Transmission Electron Microscopy (TEM)

The duodenum tissue was fixed in 2.5% glutaraldehyde under 4 °C for 24 h. The fixed tissues were washed three times with 0.1 M PBS (pH 7.4) for 15 min. The duodenum specimens were adjusted to approximately 1 mm^3^ in size, incubated in 1% osmium with 0.1 M PBS (pH 7.4) at room temperature (20 °C) for 2 h, and washed three times for 15 min with 0.1 M PBS (pH 7.4). After undergoing alcohol dehydration in concentrations of 50%, 70%, 80%, 90%, 95%, 100%, and 100% for 15 min each, the samples were permeated in an acetone-Epon 812 mixture (1:1) overnight and then were permeated in pure 812 embedding agent overnight. Once permeated, samples underwent polymerization at 60 °C for 48 h and were then cut into 60–80-nm thin sections. Uranium lead double-staining was performed by staining each section with a 2% uranium acetate saturated aqueous solution and lead citrate for 15 min/section, and then sections were dried at room temperature overnight. The intestinal mucosal barrier was observed under a JEM-1200 transmission electron microscope (Jeol, Tokyo, Japan), and each image was captured at a magnification of 8000×.

### 2.6. Real-Time PCR Assay and ELISA Assays

Total RNA from duodenum tissue was extracted by TRIzol reagent (TaKaRa, Dalian, China), and the concentration and purity of RNA were determined by a NanoDrop ND-1000 (Thermo Fisher Scientific, Waltham, MA, USA). The A260/280 ratio ranged from 1.90 to 2.00, indicating a good-quality sample. PrimeScript™ RT reagent Kit with gDNA Eraser (TaKaRa) and TB Green™ Premix Ex Taq™ II kit (TliRNaseH Plus, TaKaRa) were used to synthesize cDNA and analyze the expression levels of IL-1β (Foward: 5′-ACAAAGCCAGAGTCCTTCAGAGA, Reverse: 5′-GCCACTCCTTCTGT GACTCCA-3′), IL-6 (Foward: 5′CTGCAAGAGACTTCCATCCAGTT-3′, Reverse: 5′-GAAGTAGGGAAGGCCGTGG-3′), TNF-α (Foward: 5′-GCGACGTGGAACTGGCA GAAG-3′, Reverse: 5′-AGAA GGTGACTCGGTCCGCATAG-3′), MPO (Foward: 5′-ACGCCTGGAGTCAATCGCAATG-3′; Reverse: 5′-ACGCTCCTGGTCCTTGGT CAG-3′), COX-2 (Foward: 5′-GGTGCCTGGTCTGATGATGTATGC-3′; Reverse: 5′-GGATGCTCCTGCTTGAGTATGTCG-3′), GAPDH (Foward: 5′-GGTGGTGCTAA GCGTGTCAT-3′, Reverse: 5′-CCCTCCACAATGCCAAAGTT-3′) according to the manufacturer’s instructions. The duodenum was made into homogenization to assess the sIgA levels according to enzyme-linked immunosorbent assay (ELISA) kit instructions (Elabscience Biotechnology, Jiangsu, China).

### 2.7. PAS Staining

Sections were soaked in 1% periodate for 10 min and washed with distilled water for 2 min. The sections were treated with Schiff’s reagent (Bohu Biotechnology Co., Ltd., Shanghai, China) in a dropwise manner and incubated for 20 min at room temperature in the dark, following which the sections were rinsed twice for 5 min under running water, re-stained with hematoxylin(Sigma-Aldrich, Shanghai, China) for 2 min, rinsed with tap water until the nuclei turned blue, dehydrated in anhydrous ethanol, cleared in xylene, air-dried in a fume hood, and sealed with neutral gum and examined microscopically. Three selected sections with better colored tissue in each sample were imaged, and viewed at 400× field of view under a light microscope. Five fields were randomly selected from each section, and the number of cup cells per 200 absorbed cells were counted and the average calculated.

### 2.8. Immunofluorescence

Paraffin sections were dewaxed in xylene I, II, and III for 10 min each and rehydrated using an alcohol gradient (100%, 100%, 95%, 90%, 80%, and 70% for 5 min each) and then distilled water for 2 min. Slides were boiled in 1% citrate antigen repair solution for 10–15 min (pH 6.0). When the samples cooled to room temperature, washed with 1× PBS 3 times at 5 min each. Sections were sealed with goat serum for 30 min at room temperature, and then incubated with rabbit antibodies against MUC-2 (1:100; cat. no. A14659; Abcam, Cambridge, UK) overnight at 4 °C in a humidified box. Next day, the sections were washed thoroughly with 1× PBS (3 times, 5 min each), and then incubated in fluorescent second antibody (cat. no. BA1032; Boster Biological Technology., LTD, Wuhan, China) at 37 °C for 30 min. After repeated washing (1× PBS, 3 times, 5 min each), the samples underwent DAPI color development for 5 min. They were then washed thoroughly (1× PBS, 4 times, 5 min each). Then, the slices were sealed with a blocking solution containing an anti-fluorescence quencher and then images were collected under a fluorescent microscope (Olympus BX53, Olympus Corporation, Tokyo, Japan).

### 2.9. DNA Extraction and Hiseq Sequencing

Microbial DNA was extracted from cecum contents using a QIAamp Fast DNA Stool Mini Kit (Qiagen, Nasdaq, New York, USA) according to the manufacturer’s instructions [33]. After the concentration and integrity of the DNA was assessed, the V3-V4 region of the 16S rRNA gene was amplified using the bacterial universal primers 341F: 5′-CCTACACGACGCTCTTCCGATCTN(barcode)CCTACGGGNGGCWGCAG-3′, 805R: 5′-GACTGGAGTTCCTTGGCACCCGAGAATTCCAGACTACHVGGG TATCTAATC C-3′. The PCR products were recovered via agarose gel electrophoresis and purified using an AxyPrep DNA Gel Extraction Kit (Axygen, Hangzhou, China) according to the manufacturer’s instructions, following which a library was constructed and sequenced on the HiSeq 2500 sequencing platform (Illumina, San Diego, CA, USA) according to the Sequencing Methods manual [34].

### 2.10. 16S rRNA Bioinformatics Analysis and Statistics

Single-end reads were assigned to samples based on their unique barcodes, and the barcodes and primer sequences were removed. Quality filtering on the raw reads was performed under specific filtering conditions to obtain high-quality clean reads according to Cutadapt [35] quality control process. The reads were compared with the reference database [36] using the UCHIME algorithm [37] to detect chimera sequences, which were subsequently removed [38] and the clean reads were obtained.

Species annotation analysis was performed using the Mothur method for OTUs with 97% similarity (setting a threshold of 0.8 to 1), and the abundance of OTUs was calculated. Subsequent alpha diversity and beta diversity analyses were performed based on homogenized OTU abundance information, using the sequence number criterion corresponding to the sample with the smallest sequence [39]. To analyze the complexity of species diversity, OTUs with 97% similarity were analyzed using QIIME software (Version 1.9.1) to calculate alpha diversity indices, including Chao1, ACE (community richness), Shannon, Simpson (diversity), and Good s coverage (sequencing depth) [40]. Weighted UniFrac distances were calculated using Qiime software (Version 1.7.0) and PCoA plots were drawn using R software (Version 2.15.3) to obtain and visualize principal coordinates from complex multidimensional data. The Bray–Curtis algorithm was used to construct the UPGMA sample clustering trees. The distance matrix was interpreted and combined with bacterial community histograms to evaluate microbial taxon similarities and taxonomic differences between taxa or samples [41]. We performed linear discriminant analysis (LDA) with LEFSE, with a default set LDA score filter value of 4, to identify species that were significantly different between groups, while statistical significance was determined using one-way analysis of variance (ANOVA) followed by Tukey’s test. All statistical tests were conducted using Prism software (Prism 6.0; GraphPad Software, San Diego, CA, USA). Statistical significance was set at *p* < 0.05. Sequencing services, database construction, and statistical analyses were carried out by Beijing Noggin Technology Co., Ltd. (Beijing, China) [33].

## 3. Results

### 3.1. Histopathological Changes of Duodenum

**Figure 1:**
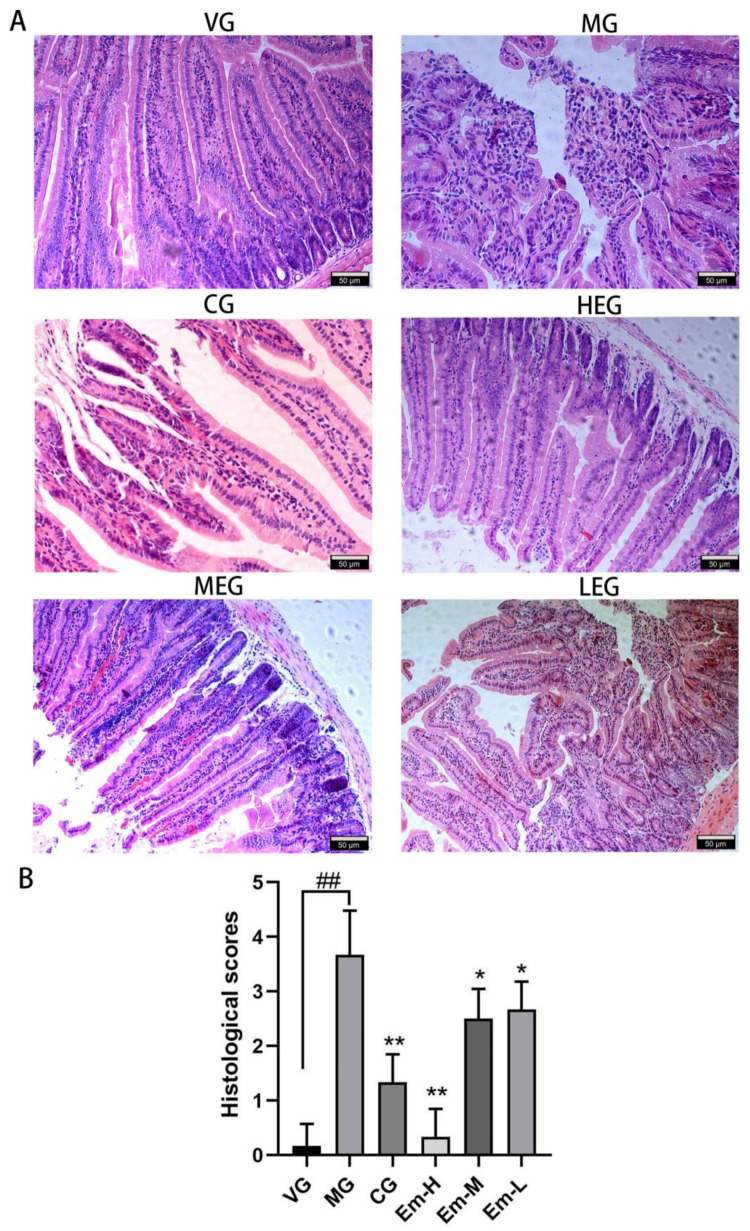
Effects of emodin on intestinal injury in the duodenum of mice with *E. coli* O_1_-induced diarrhea. (**A**) Representative hematoxylin-eosin-stained sections showing the morphology of the duodenum in the different treatment groups: the vehicle group (VG), model group (MG), ciprofloxacin treatment group (CG), high-dose emodin group (Em-H), middle-dose emodin group (Em-M), and low-dose emodin group (Em-L). The magnification is 200×. (**B**) Histological scores based on Chiu’s scale for the VG, MG, CG, Em-H, Em-M, and Em-L groups. Values are means, and their standard errors are visualized using vertical error bars (*n* = 8). ## = *p* < 0.05, significant between VG and MG alone; * = *p* < 0.05; ** = *p* < 0.01; and ns = *p* = 0.05, significant between treatment group and MG.

As observed by light microscopy (Figure 1), mice in the VG group maintained intact intestinal mucosa and showed neatly arranged intestinal villi. In contrast, the intestinal mucosa from MG mice that received intragastric administration of *E. coli* O_1_ showed conspicuous damage, which was characterized by intestinal edema, a large inflammatory cell infiltration, intestinal villi disintegration and loss of contour (Figure 1A). The MG group showed a high pathological score that was significantly different from that of the VG group (*p* < 0.05, Figure 1B). Mice treated with emodin or with ciprofloxacin (CG) showed suffused inflammatory infiltration without distinct intestinal necrosis and hemorrhage (Figure 1A) compared to the MG group. Further, the pathological severity scores of the mice in the CG and emodin treatment groups were lower than those of the MG mice (*p* < 0.05, Figure 1B). These results demonstrate that treatment with emodin may alleviate pathological damage to the intestinal mucosa in mice with *E. coli* O_1_-induced diarrhea. 

### 3.2. Ultrastructural Changes in Duodenum

**Figure 2:**
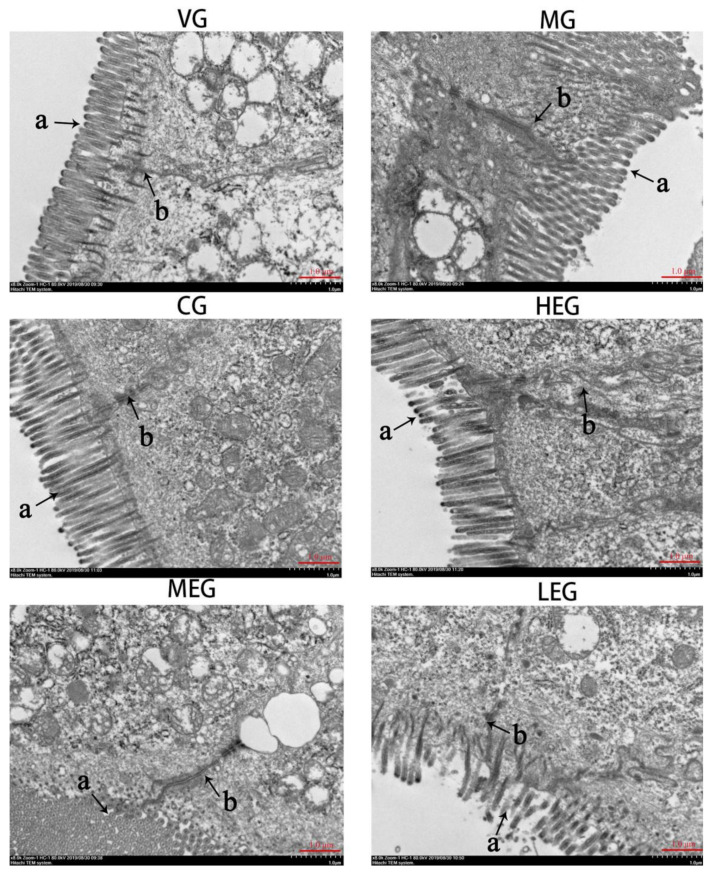
Effects of emodin on intestinal ultrastructural injury in the duodenum of mice with *E. coli* O_1_-induced diarrhea (8000×). Representative micrographs of the vehicle group (VG), model group (MG), ciprofloxacin treatment group (CG), high-dose emodin group (Em-H), middle-dose emodin group (Em-M), and low-dose emodin group (Em-L). The small letter “a” in the diagram represents intestinal microvilli, while “b” represents tight junction.

As observed by TEM (Figure 2), the VG group showed clear cellular junctions with closely arranged intestinal epithelial cells, and the microvilli on the surface of the epithelial cells were neatly arranged, tight and of uniform length beside the junctional complex being structurally normal. However, in the MG group, the microvilli on the surface of the intestinal epithelial cells were sparse and atrophic with irregular lengths, accompanied by variable degrees of shedding. Moreover, the tight junctions between cells markedly widened. Mice in the Em-H and CG groups showed tightly connected epithelial cells and relatively clear and normal microvilli structures compared to the MG group. These results indicate that a high-dose of emodin alleviated pathological changes in the intestinal TJ barrier of mice with *E. coli* O_1_-induced diarrhea.

### 3.3. Cytokine Changes in Duodenum

**Figure 3:**
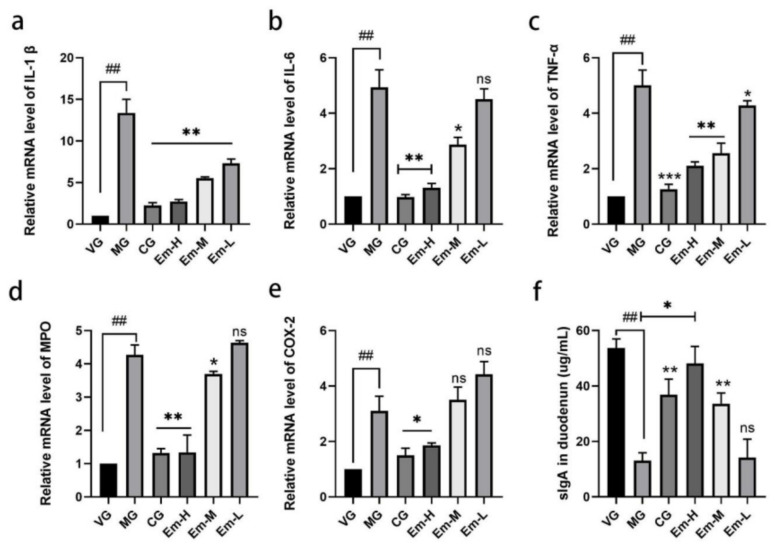
Effects of emodin on mRNA levels IL-1β (**a**), IL-6 (**b**), TNF-α (**c**), MPO (**d**), and COX-2 (**e**) levels and sIgA (**f**) level in the duodenum of mice with *E. coli* O_1_-induced diarrhea for the vehicle group (VG), model group (MG), ciprofloxacin treatment group (CG), high-dose emodin group (Em-H), middle-dose emodin group (Em-M), and low-dose emodin group (Em-L). Data are expressed as means ± SD. Values are means, and their standard errors are visualized using vertical error bars (*n* = 8). ## *= p* < 0.05, significant between VG and MG alone; * = *p* < 0.05; ** = *p* < 0.01; *** = *p* < 0.01 and ns = *p =* 0.05, significant between treatment group and MG.

As shown in Figure 3, mRNA levels of IL-1β, IL-6, TNF-α, MPO, and COX-2 were strikingly increased in the MG compared to those in the VG group (*p* < 0.01). These levels were particularly reduced in the CG (*p* < 0.05) and Em-H groups (*p* < 0.01) compared to those in the MG group. Moreover, the serum level of sIgA in MG was strikingly decreased compared to VG, and CG (*p* < 0.05) and Em-H could have contributed to this increase.

### 3.4. Changes in Duodenal Goblet Cells

**Figure 4:**
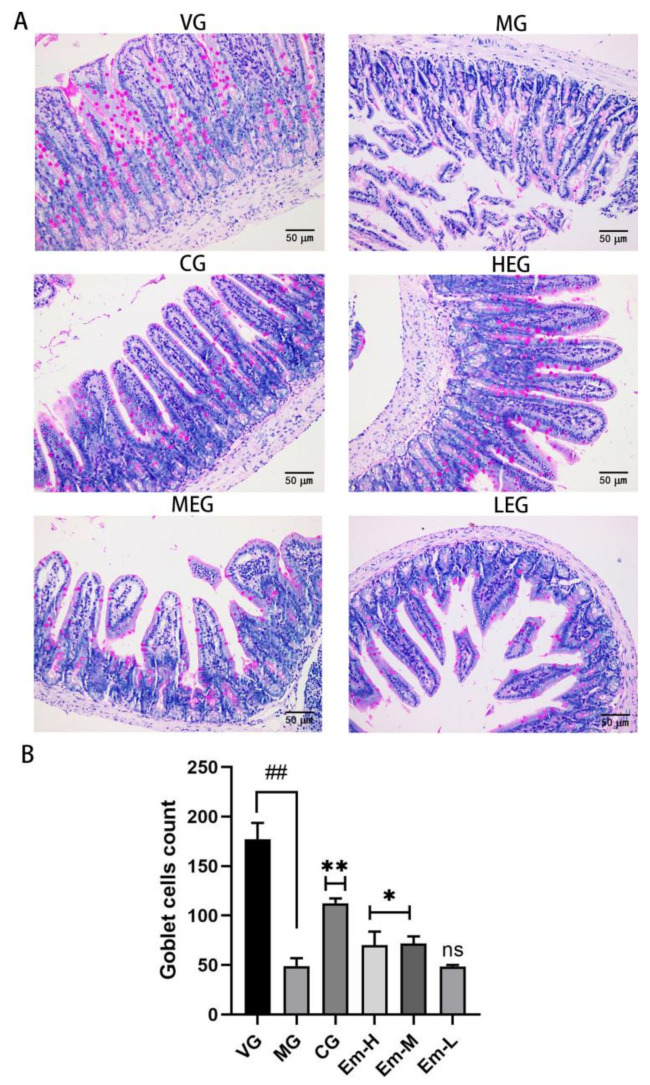
Effects of emodin on morphology and number of duodenum epithelial goblet cells in mice with *E. coli* O_1_-induced diarrhea. (**A**) Representative periodic acid-Schiff-stained sections showing the morphology of goblet cells in the different treatment groups: the vehicle group (VG); the model group (MG); the ciprofloxacin treatment group (CG); the high-dose emodin group (Em-H); the middle-dose emodin group; (Em-M), and the low-dose emodin group (Em-L). The magnification is 400×. (**B**) Goblet cell count for the MG, Em-H, Em-M, and Em-L groups.Values are means, and their standard errors are visualized using vertical error bars (*n* = 8). ## *= p* < 0.05, significant between VG and MG alone; * = *p* < 0.05; ** = *p* < 0.01; and ns = *p =* 0.05, significant between treatment group and MG.

PAS staining (Figure 4), revealed that VG duodenum intestinal mucosal cupped cells were round or oval in shape, numerous, clearly colored, and densely distributed in all parts of the villi, with color intensity changing from dark at the base of the villi to light at the top of the villi. However, the MG duodenum intestinal mucosa had fewer cupped cells, with indistinct cell coloration and sporadic distribution throughout the intestinal villi. Compared with MG, mice in the Em-H, Em-M, and CG groups showed significantly increased numbers of goblet cells.

Duodenum intestinal villous cup cells, which were clearly colored and densely distributed in all parts of the intestinal villi (Figure 4A). Compared to VG, the number of duodenal cupped cells was significantly lower in MG (*p* < 0.05, Figure 4B). However, compared to MG, duodenal cupped cells were significantly increased in all treatment groups except for Em-L (*p* < 0.05; *p* < 0.01; Figure 4B).

### 3.5. Changes of MUC-2 in the Duodenum

**Figure 5:**
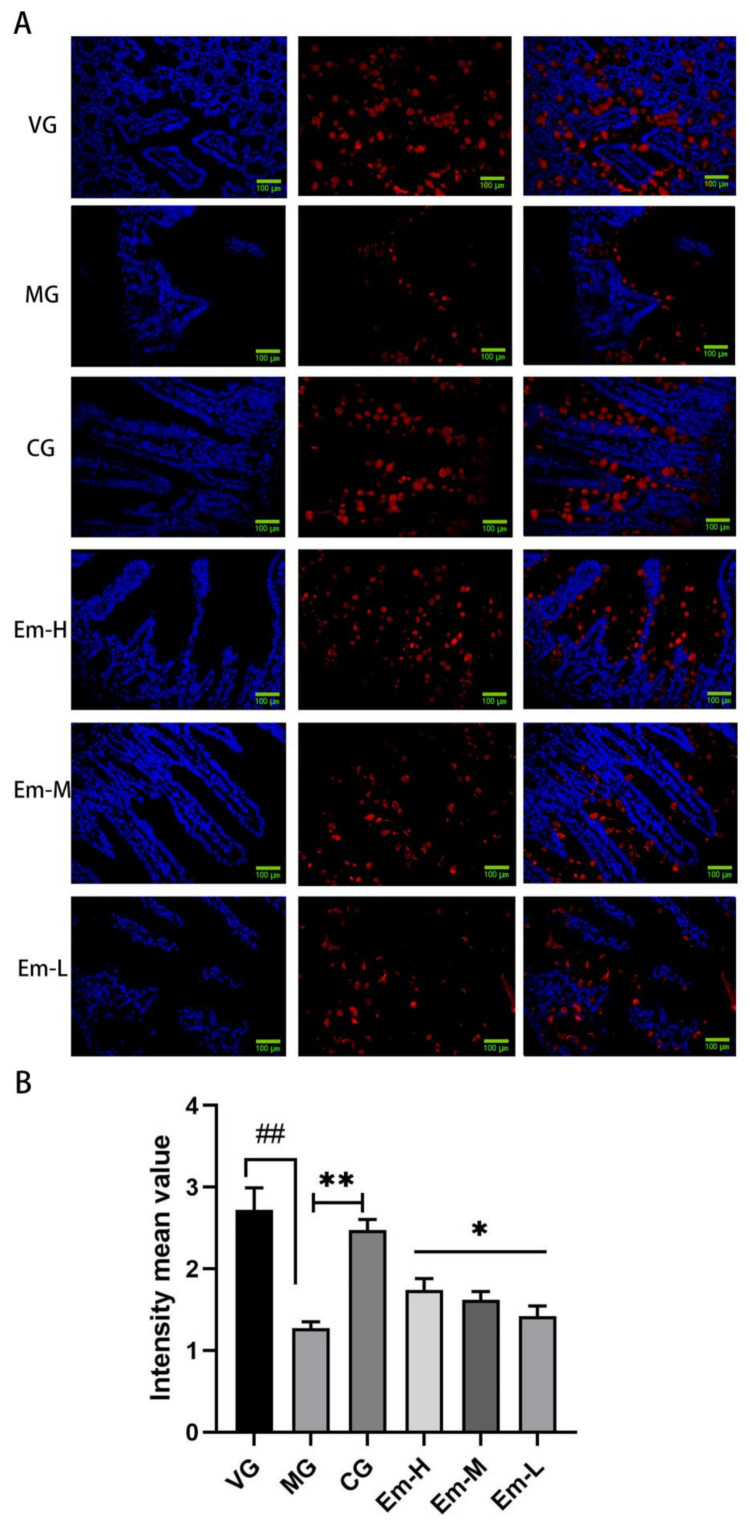
Effects of emodin on MUC-2 secretion by the duodenal epithelium in mice with *E. coli* O_1_-induced diarrhea. (**A**) Representative immunofluorescence sections showing MUC-2 secretion by the duodenal epithelium. (**B**) Intensity mean value for the MG, Em-H, Em-M, and Em-L groups. Values are means, and their standard errors are visualized using vertical error bars (*n* = 8). ## *= p* < 0.05, significant between VG and MG alone; * = *p* < 0.05; ** = *p* < 0.01; and ns = *p* = 0.05, significant between treatment group and MG.

Immunofluorescence (Figure 5) showed that the fluorescence intensity of MUC-2 in the duodenum of MG was significantly weaker than that of VG (*p* < 0.01). However, compared to that of MG, the fluorescence intensity of MUC-2 was significantly enhanced in mice treated with emodin or ciprofloxacin (CG) (*p* < 0.05).

### 3.6. Changes in Intestinal Microbiota

#### 3.6.1. Alpha Diversity Analysis

The observed species index and Goods coverage index are indices of sequencing depth. A sequencing depth of 98% to 99% completely covers the species that are likely to be present in a sample. To better understand the OTU diversity of each group, we used Chao 1, ACE, as well as Simpson and Shannon indices to estimate the abundance and diversity of bacterial communities in the cecum contents of different groups of mice, respectively, and compared alpha diversity. Community diversity was reflected by Shannon and Simpson, and community richness was reflected by Chao1 and ACE, where VG had the highest Shannon, Chao1, and ACE levels, while MG had the lowest level. However, Shannon, Chao1, and ACE indices were significantly higher in MG than in the emodin-treated group, and there were no significant differences with the VG group (Table 1).

#### 3.6.2. Beta Diversity Analysis

**Figure 6:**
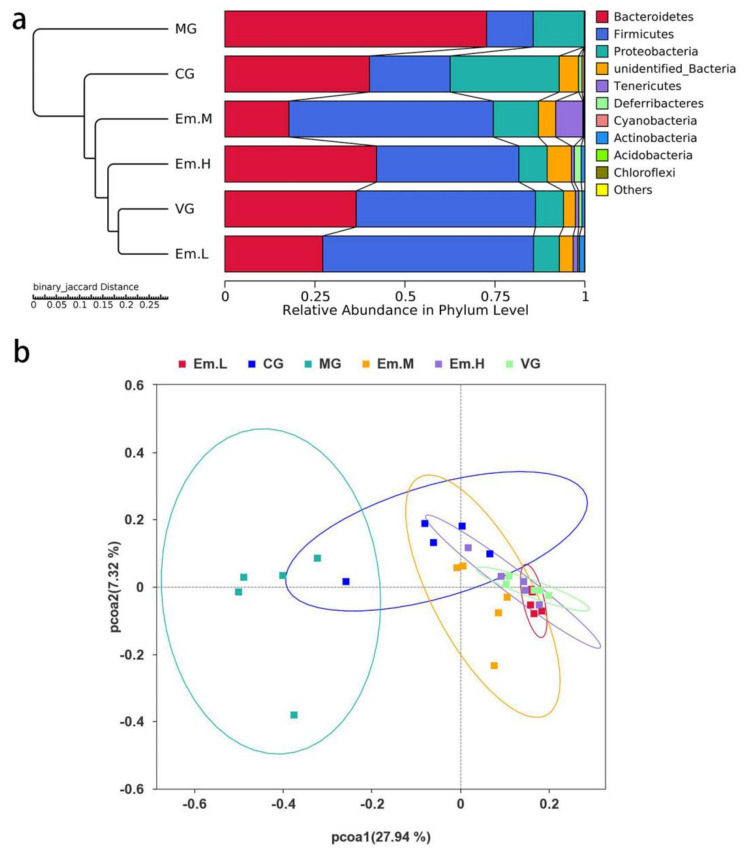
Effects of emodin on gut microbiota structure of cecum microbial community in mice with *E. coli* O_1_—induced diarrhea. (**a**) UPGMA clustering tree and (**b**) PCoA score plot are based on binary-Jaccard distances in the different treatment groups: the vehicle group (VG); the model group (MG); the ciprofloxacin treatment group (CG); the high—dose emodin group (Em.H); the middle—dose emodin group (Em.M); and the low—dose emodin group (Em.L).

PCoA and UPGMA clustering tree based on binary-Jaccard distances were used to analyze changes in the overall structure of gut microbiota (Figure 6). Both PCoA and UPGMA showed an independent distribution of intestinal microbial community in MG and CG, indicating that mouse modeling and antibiotic treatment may significantly change the intestinal microbiota structure. By contrast, the VG and Em treatment groups showed no obvious independent distribution and some areas overlapped, suggesting that Em treatment restored the disturbed microbiota caused by modeling and was similar to the intestinal microbiota of VG.

#### 3.6.3. Taxonomic Composition of Intestinal Microbiota

At the phylum level (Table 2), the four most abundant phyla in the cecal luminal contents in each group were *Firmicutes*, *Proteobacteria*, *Bacteroidetes*, and *Verrucomicrobia*, Within VG samples, *Bacteroidetes* (93%), *Firmicutes* (82%), *Verrucomicrobia* (23%), and *Proteobacteria* (11%) were more abundant, whereas the relative abundances in cecal luminal contents of MG samples were significantly different, with *Bacteroidetes*(9%), *Firmicutes* (24%), and *Verrucomicrobia* (5%) significantly reduced, and *Proteobacteria* (63%) significantly increased. Emodin and antibiotic treatment groups enriched *Bacteroidetes* (27–92%), *Firmicutes* (30–70%), and *Verrucomicrobia* (1%, 15%) while inhibiting *Proteobacteria* (7–56%), when compared with that in the MG group (*p* < 0.05).

At the order level (Table 3), the five most abundant orders of cecal luminal contents in each group were *Bacteroidetes*, *Clostridiales*, *Enterobacteriales*, *Lactobacillus*, and *Verrucomicrobiales*, where *Bacteroidetes* (85%), *Lactobacillus* (33%), and *Verrucomicrobiales* (23%) were more abundant than the other two. Whereas the relative abundances in the cecal luminal contents of MG samples were significantly different, with *Clostridiales* (67%) and *Enterobacteriales* (35%) significantly increased and *Bacteroidetes* (4%), *Lactobacillus* (3%) and *Verrucomicrobiales* (5%) significantly reduced, In the emodin and antibiotics treatment group, *Bacteroidetes* (22–45%), *Lactobacillus* (5%, 15%), and *Verrucomicrobiales* (15%, 11%) were enriched, whereas *Clostridiales* (26–53%) and *Enterobacteriales* (1–11%) were inhibited compared with that in the MG (*p* < 0.05).

#### 3.6.4. LEFse Analysis

**Figure 7:**
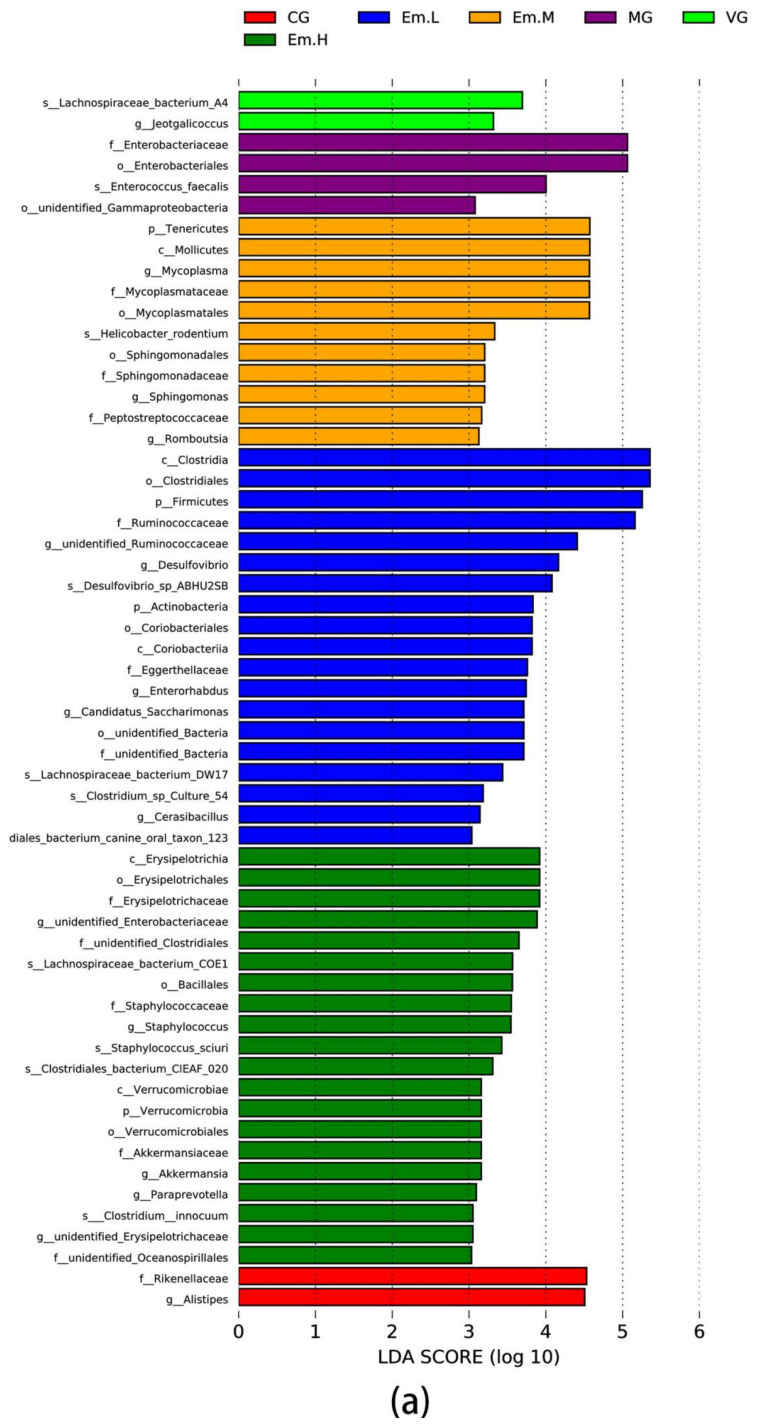

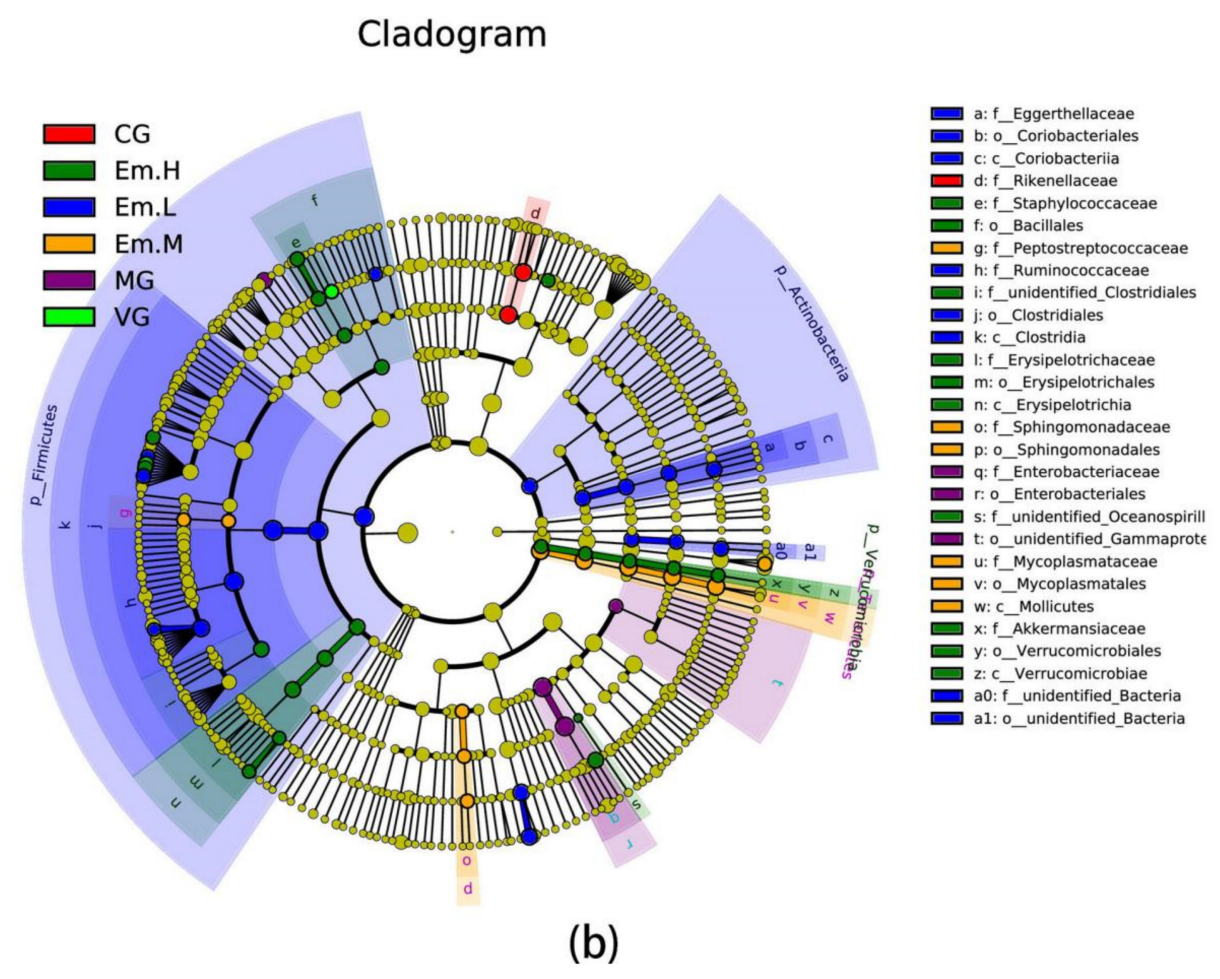
Analysis of between group differences in the dominant microorganisms of cecum bacteria using LEfSe. (**a**) Histogram showing the LDA scores used to determine dominant taxa based on OTU tables. Differently colored histograms represent different groups. (**b**) Diagram of the evolutionary branches; the circles radiating from the inside to outside represent taxonomic levels from phylum to genus (or species).

The LEfSe analysis of intestinal microbiota is shown (Figure 7). The results showed that *Lachnospiraceae_bacterium_A4* was more dominant in dominant microbiota, whereas *Enterobacteriaceae*, *Enterococcus*, and *Gammaproteobacteria* were the dominant communities in MG. However, compared to MG, 20 differential taxa were found in the Em-H group, Among these, class Erysipelotrichia, order Erysipelotrichales, and family *Erysipelotrichaceae* showed dominance, in addition to which, phyla Verrucomicrobia, class Verrucomicrobiae and family *Akkermansiaceae*, genus *Akkermansia* also played an important role. Eleven dominant taxa were found in the Em-M group. Among these, phyla Tenericutes, class Mollicutes, genus *Mycoplasma*, family *Mycoplasmataceae*; order Mycoplasmatales showed dominance. There are twenty differential taxa were found in the Em-L group, among these, phyla Firmicutes, class Clostridia, order Clostridiales, family *Ruminococcaceae* showed dominance. Furthermore, family *Rikenellaceae*, genus *Alistipes* played an important role in CG.

## 4. Discussion

*E. coli*-induced diarrhea is an acute inflammatory disease, which is causing widespread concern due to increased incidence, rapid morbidity, and mortality. However, there is a lack of environmentally acceptable and safe drugs for treatment. Results of the present study indicates that emodin attenuates the pathogenic *E. coli*-induced intestinal inflammatory response, which is associated with the regulation of intestinal microbiota.

The morphological structure of the intestinal mucosa is an important barrier that protects the body from pathogenic microorganisms and is an important indicator of the health and functional recovery of the intestine, which is used in the diagnosis of gastrointestinal diseases [42,43]. Liu et al., reported that *E. coli* colonized the small intestine and disrupted intestinal barrier function by releasing specific enterotoxins, which stimulate the secretion of pro-inflammatory cytokines IL-1β, IL-6, and TNF-α, leading to diarrhea and even death [44]. Ren et al., showed that ETEC infection promotes the expression of pro-inflammatory cytokines via activation of the NF-kB and MAPK pathways, leading to the absence of jejunal microvilli [18]. In addition, Liu et al. showed that the absorption of emodin was significantly higher in the duodenum than in the ileum and colon in the rat isolated intestinal perfusion model. So, in the present study, we examined the effect of emodin on duodenum [45]. Data shows that ETEC infection resulted in the breakage and irregular arrangement of intestinal villi as well as microvilli loss. Moreover, tight junction gaps in the duodenal tissue of the MG were widened, along with a significant increase in the expression of inflammatory factors, including IL-1β, IL-6, TNF-α, COX-2, and MPO. These results are consistent with those of the previous study. However, treatment with emodin attenuated the effect of ETEC challenge to duodenal morphology, as indicated by the relatively low intestinal histological scores and inflammatory factor gene expression in mice after emodin administration. In addition, emodin exerts a strong antibacterial effect [46]. Therefore, we speculated that emodin may improve the prognosis of *E. coli* related diarrhea by repairing the intestinal barrier function via its antibacterial and anti-inflammatory effects. Further studies are required to elucidate the specific mechanisms underlying its action.

ETEC infections are often associated with diarrhea and impaired intestinal barrier function. Goblet cells are an important component of the non-specific intestinal barrier that protects animals from pathogenic bacteria [47]. Mucin, predominantly mucin-2 (MUC-2), is secreted by the goblet cells, which form the intestinal mucus system and effectively prevents microbial penetration into the intestinal epithelium and crypts. A decrease in the number of goblet cells may reduce mucin secretion in the mucosa, which exerts a detrimental effect on the mucosal barrier [48]. In the current study, emodin increased the number of epithelial goblet cells and MUC-2 expression levels in the duodenum of pathogenic *E. coli* O_1_-infected mice. This suggested that emodin exerted an ameliorative effect on the mucosal barrier of mice affected by *E. coli* O_1_ related diarrhea. Reportedly, sIgA, which is the main antibody isotype in the intestinal mucosa, plays a vital role in the intestinal mucosal barrier [49]. SIgA prevents bacteria from binding to epithelial cell receptors and stimulates intestinally secreted mucus, accelerates the flow of the mucus layer, and effectively prevents bacteria from adhering to the intestinal mucosa [50]. In our study, emodin increased the levels of SIgA in the duodenum of pathogenic *E. coli* O_1_-infected mice. These results suggest that emodin could increase sIgA content by preventing *E. coli* from adhering to the intestinal epithelium, thereby relieving diarrhea in mice.

The association between intestinal microbiota and diarrhea has been extensively studied [51]. Gut microbiota plays an important role in the development of the host immune system and the intestinal mucosa [52]. In this study, we used Illumina HiSeq to barcode the 16S rRNA V3-V4 high-variant region to compare the microbiota composition in the cecum of pathogenic *E. coli* O_1_-induced diarrheal mice and emodin treated mice. Chao1 and ACE analysis showed that microbial diversity was higher in VG than in MG, as confirmed by Shannon and Simpson indices (which also showed that bacterial community diversity was higher in VG than in MG). This indicated that, *E. coli* O_1_ infection reduces the diversity and richness of intestinal microbiota in mice. Further, Sun et al., concluded that *E. coli* O_101_ infection reduced the diversity of gut microbiota in rats [11]. Thus, our findings are consistent with those of previous studies. However, following emodin treatment, bacterial community richness and diversity in the diarrhea affected mice were increased, indicating that emodin prevented the dysbiosis of gut microbiota caused by *E. coli* O_1_. UPGMA clustering analysis showed that the microbiota of mice in the VG and EM groups clustered together, while the cecum microbiota of MG and CG were separated. Such clustering may depend on *E. coli* strain and drug treatment, where PCoA analyses suggest that a differentiated and independent distribution existed in the model between antibiotic treatment and emodin groups, with a shorter distance between the vehicle group and the emodin group, where the results of UPGMA and PCoA showed that the structure of the intestinal microbiota was significantly altered by *E. coli* infection and antibiotic treatment, while emodin treatment corrected the disturbances in the intestinal microbiota.

In this study, we analyzed the dynamics of the intestinal microbiota. At the phylum level, Bacteroidetes Firmicutes, Proteobacteria, and Verrucomicrobia were the most abundant in each group, which was in agreement with the findings of a previous study [53]. However, the dominance of these microbiota changed following *E. coli* O_1_ infection. The relative abundance of Bacteroidetes, Firmicutes and Verrucomicrobia decreased significantly, while the relative abundance of Proteobacteria increased significantly. Compared with that of the model group, emodin treatment increased the ratio of Bacteroidetes, Firmicutes and Verrucomicrobia and decreased the relative abundance of Proteobacteria. These data demonstrated that emodin prevented the dysbiosis of intestinal microbiota caused by *E. coli* O_1_. Recent studies have shown that diarrhea caused by *E. coli* was associated with a decrease in the ratio of the phyla, Bacteroidetes and Firmicutes, and an increase in the ratio of Actinobacteria [54]. Sun et al., demonstrated that *E. coli* O_101_ infection decreased the abundance of Firmicutes and Verrucomicrobia and increased the abundance of Proteobacteria and Actinobacteria [11]. Our findings were consistent with those of previous studies.

Changes at the order level were generally consistent with those at the phylum level in this study (i.e., in the model group, the abundance of Clostridiales and Enterobacteriales were significantly increased, while that of Bacteroidetes, Lactobacilliales, and Verrucomicrobiales were significantly reduced). Enterobacteriales belongs to Proteobacteria while *Lactobacillus* belongs to Firmicutes. The abundance of *Clostridiales* and *Enterobacteriales* in the emodin treatment group were found to be decreased, while that of *Bacteroidetes and Verrucomicrobiales* were found to be increased. These data suggested that emodin inhibits the growth of harmful bacteria and promotes the growth of beneficial bacteria, and that *Lactobacillus* and *Enterobacteriales* may be the main genera contributing to changes in *Firmicutes* and *Proteobacteria* following *E. coli* O_1_ infection. Lactobacillus is an indispensable beneficial bacterium in the intestinal tract, which not only exerts antimicrobial effects through the secretion of lactic acid, hydrogen peroxide, and bacteriocins, but also improves intestinal barrier function by competing for adhesion sites and regulating immune inflammation [55]. Enterobacteriaceae is mainly characterized by endotoxin production, which is closely related to metabolic endotoxin levels and systemic inflammation [56]. In the present study, the relative abundance of *Enterobacteriales* was positively correlated with inflammatory markers and plasma endotoxin levels, which is presumed to be related to the inhibitory effect of emodin on endotoxin-producing bacteria, especially *Enterobacteriales*. However, there is still a need to analyze the intestinal flora selectively enriched or inhibited by emodin at higher concentrations.

We used LEfSe analysis to identify the differential microbial taxa most likely to cause differences between groups. The data showed that the main species that differed between the model and healthy groups were Enterobacteriaceae, *Enterococcus*, Enterobacteriales, and Gammaproteobacteria, all of which belong to phylum Proteobacteria. The results showed that the *E. coli*-associated and dominant Proteobacteria was the main microorganism responsible for diarrhea and inflammation of the intestine in mice. Phylum Verrucomicrobia, Class Verrucomicrobiae, family Akkermansiaceae, genus *Akkermansia* also played an important role in the emodin treatment group. Studies have shown that the abundance of *Akkermansia* is significantly lower in the feces of pigs with epidemic diarrhea [57]. Abundance of Verrucomicrobiae was also reduced in the osmotic diarrhea mouse microbiota [58]. Results obtained in our study were consistent with those of previous studies, and we speculated that emodin protects the intestinal barrier by improving intestinal microbial community in diarrhetic mice. Verrucomicrobia and *Akkermansia* are closely associated with diarrhea and the intestinal mucus barrier. Verrucomicrobia is mainly concentrated in the mucus layer of the intestine and is considered to be an indicator of a healthy intestine due to its immunostimulatory properties and ability to improve the intestinal barrier [59]. *Akkermansia* is a mucin-degrading bacterium that resides in the mucus layer and has the ability to stimulate mucin synthesis, restore the thickness of the mucus layer and the number of cupped cells, and thereby improve the barrier function of the intestinal mucus layer [60]. *Akkermansia* also has a strong anti-inflammatory effect, mainly due to its ability to increase endogenous cannabinoids in the intestinal tract, and thereby controls intestinal barrier integrity, intestinal peptide hormone secretion, and inflammation levels [61]. In addition, the order and family that *Erysipelotrichia* belong to were mainly found in the high-dose emodin treatment group. It has been shown that the enrichment of *Erysipelotrichia* is positively correlated with the formation of a stronger mucus layer of the intestinal barrier, and certain species belonging to are associated with the formation and properties of intestinal mucus [60]. Thus, the protective effect of emodin on the intestinal tract may also be related to the increased abundance of *Erysipelotrichia*. Considered together with the previously described results of higher goblet cell counts and MUC-2 expression levels in the high-dose emodin treatment group compared to that of the model control group, these findings suggested that emodin improves diarrhea by increasing levels of the intestinal barrier-protecting bacteria, *Verrucomicrobia*, *Erysipelotrichia*, and *Akkermansia*, thereby repairing the mucus barrier and protecting the intestine.

Rhubarb is grown in many regions in China, with high production. As the most important active ingredient of the herb rhubarb, emodin has a wide range of applications, such as an antibacterial agent [62], in the form of a broad-spectrum antibacterial drug, as well as a food additive. Its anti-inflammatory properties [63] can be exploited in feed additives; its anti-tumor properties [64] can be exploited in cancer treatment and prevention; and its antiviral properties can facilitate the development of new generation antiviral drugs, which could be applied in the management of human and animal epidemics [65]. Furthermore, its positive effects on the gastrointestinal tract can be exploited to develop healthcare products for enhancing gastrointestinal development in newborn animals [66]. However, mechanisms via which emodin relieves diarrhea in animals (e.g., chickens, pigs) require further investigations. Overall, emodin could facilitate the improvement of animal welfare and the sustainable development of animal husbandry.

## 5. Conclusions

Our results suggested that emodin protects against intestinal damage induced by *E. coli* O_1_ and strengthens the intestinal mucosal barrier function in mice by increasing the abundance of beneficial intestinal microbiota and inhibiting the abundance of harmful bacteria, thereby alleviating diarrhea.

## Figures and Tables

**Table 1:** Alpha diversity index for each group.

Group	Community Diversity		Community Richness			
	Shannon	Simpon	Chao1	ACE	Observed_Species	Goods_Coverage
VG	6.36 ± 0.18 ^a^	0.44 ± 0.12 ^c^	524.52 ± 44.31 ^a^	515.31 ± 18.84 ^a^	463.60 ± 19.68 ^a^	0.998 ± 0.0002
MG	1.70 ± 0.18 ^c^	0.96 ± 0.13 ^a^	216.56 ± 16.88 ^c^	233.26 ± 14.09 ^c^	174.80 ± 16.52^c^	0.998 ± 0.0002
CG	4.11 ± 0.45 ^b^	0.87 ± 0.08 ^b^	370.63 ± 41.56 ^b^	386.65 ± 15.44 ^b^	325.40 ± 18.38 ^b^	0.998 ± 0.0002
Em-H	6.03 ± 0.35 ^a^	0.40 ± 0.06 ^c^	527.11 ± 27.30 ^a^	521.03 ± 23.90 ^a^	460.00 ± 26.04 ^a^	0.998 ± 0.0002
Em-M	5.56 ± 0.31 ^a^	0.46 ± 0.04 ^c^	487.61 ± 36.78 ^a^	476.59 ± 37.01 ^a^	412.80 ± 37.08 ^a^	0.998 ± 0.0002
Em-L	6.45 ± 0.28 ^a^	0.46 ± 0.09 ^c^	566.27 ± 24.78 ^a^	550.91 ± 21.23 ^a^	503.40 ± 18.42 ^a^	0.998 ± 0.0002

Note: Data are expressed as mean ± standard deviation (*n* = 8); values in same column with different letter superscripts indicate significant differences (*p* < 0.05), while the same letter superscripts indicate no significant difference (*p* > 0.05).

**Table 2:** Relative abundance of bacteria on a phylum level in each group.

Relative Abundance (%)	Groups					
	VG	MG	CG	Em-H	Em-H	Em-H
*Bacteroidetes*	0.93 ± 0.04 ^a^	0.09 ± 0.07 ^c^	0.32 ± 0.05 ^b^	0.92 ± 0.04 ^a^	0.42 ± 0.16 ^b^	0.27 ± 0.01 ^c^
*Firmicutes*	0.82 ± 0.09 ^a^	0.24 ± 0.07 ^d^	0.31 ± 0.07 ^d^	0.10 ± 0.06 ^b^	0.50 ± 0.05 ^c^	0.30 ± 0.12 ^d^
*Proteobacteria*	0.11 ± 0.02 ^c,d^	0.63 ± 0.05 ^a^	0.19 ± 0.06 ^c^	0.07 ± 0.01 ^d^	0.56 ± 0.01 ^d^	0.32 ± 0.06 ^b^
*Verrucomicrobia*	0.23 ± 0.07 ^a^	0.05 ± 0.00 ^c^	0.10 ± 0.00 ^bc^	0.15 ± 0.02 ^b^	0.05 ± 0.00 ^c^	0.03 ± 0.01 ^c^

Note: Data are expressed as mean ± standard deviation (*n* = 8); values in the same column with different letter superscripts indicate significant differences (*p* < 0.05), while same letter superscripts indicate the absence of a significant difference (*p* > 0.05).

**Table 3:** Relative abundance of bacteria on an order level in each group.

Relative Abundance (%)	Groups					
	VG	MG	CG	Em-H	Em-M	Em-L
*Bacteroidetes*	0.85 ± 0.05 ^a^	0.04 ± 0.01 ^c^	0.22 ± 0.06 ^d^	0.45 ± 0.03 ^b^	0.35 ± 0.00 ^c^	0.25 ± 0.04 ^d^
*Clostridiales*	0.03 ± 0.01 ^e^	0.67 ± 0.11 ^a^	0.34 ± 0.01 ^c^	0.26 ± 0.02 ^d^	0.48 ± 0.03 ^b^	0.53 ± 0.01 ^b^
*Enterobacteriales*	0.01 ± 0.00 ^c^	0.35 ± 0.03 ^a^	0.01 ± 0.00 ^c^	0.01 ± 0.00 ^c^	0.01 ± 0.00 ^c^	0.11 ± 0.00 ^b^
*Lactobacillus*	0.33 ± 0.03 ^a^	0.03 ± 0.01 ^c^	0.15 ± 0.00 ^b^	0.05 ± 0.00 ^c^	0.03 ± 0.00 ^c^	0.02 ± 0.00 ^c^
*Verrucomicrobiales*	0.23 ± 0.07 ^a^	0.05 ± 0.00 ^c^	0.11 ± 0.00 ^bc^	0.15 ± 0.02 ^b^	0.04 ± 0.00 ^c^	0.03 ± 0.01 ^c^
*Desulfovibrionales*	0.07 ± 0.00 ^a^	0.18 ± 0.00 ^a^	0.19 ± 0.00 ^a^	0.03 ± 0.00 ^a^	0.04 ± 0.00 ^a^	0.05 ± 0.00 ^a^
*Campylobacterales*	0.03 ± 0.00 ^a^	0.05 ± 0.00 ^a^	0.00 ± 0.00 ^a^	0.06 ± 0.00 ^a^	0.04 ± 0.00 ^a^	0.03 ± 0.00 ^a^

Note: Data are expressed as mean ±standard deviation (*n* = 8); values in the same column with different letter superscripts indicate significant differences (*p* < 0.05), while the same letter superscripts indicate the absence of a significant difference (*p* > 0.05).

## Data Availability

All raw data in the current study are available from the corresponding author.
